# Inhibition of bacterial attachment to polyethersulfone membranes using aminophenol compounds in static and continuous flow systems

**DOI:** 10.1038/s41598-025-15558-9

**Published:** 2025-08-21

**Authors:** Mohamed Salah El-Din Hassouna, Nermine Nasser, Noha Salem, Ranya Amer, Sherif H. Kandil, Norhan Nady

**Affiliations:** 1 Department of Environmental Studies, Institute of Graduate Studies and Research, Alexandria University, Alexandria, Egypt; 2 Department of Environmental Biotechnology, Genetic Engineering and Biotechnology Research Institute (GEBRI), City of Scientific Research and Technological Applications (SRTA-City), Borg El-Arab City, Egypt; 3 Polymeric Materials Research Department, Advanced Technologies and New Materials Research Institute (ATNMRI), City of Scientific Research and Technological Applications (SRTA-City), New Borg El-Arab City, 21934 Alexandria Egypt; 4 Faculty of Biotechnology, October University for Modern Sciences and Arts (MSA university), Giza, Egypt; 5 Department of Materials Science, Institute of Graduate Studies and Research, Alexandria University, Alexandria, Egypt

**Keywords:** Membrane biofouling, Poly(ethersulfone), Aminophenol, Environmentally friendly modification, Laccase, Microbiology, Environmental sciences, Materials science

## Abstract

Biofouling is the most challenging problem associated with membrane-based filtration technology. Biofouling deteriorates membranes used in filtration process and decreases the efficiency of membrane productivity used in separation fields, thus increasing the technology cost of membrane-based filtration *process*. Several approaches exist to control biofouling; membrane surface modification has recently been used to resist bacterial attachment and biofilm formation. In this study, the poly(ethersulfone) (PES) membrane was modified by three different poly(aminophenol) as bacterial anti-attachment compounds. Each one of poly(2-aminophenol), poly(3-aminophenol), and 4-aminophenol oligomers, was incorporated separately. In this test, two bacterial strains; *Escherichia coli* (Gram-negative) and *Staphylococcus aureus* (Gram-positive), were used through both static and continuously flowing fluid bacterial suspension systems to evaluate the bacterial anti-attachment performance of the modified PES membranes. The results indicated that the brush-like structured layer of poly(3-aminophenol) can resist bacterial attachment under static conditions. In addition, poly(2-aminophenol) gave better impedance of bacterial attachment in the continuously flowing fluid system. This work revealed up to 90% reduction of attached bacteria on modified membranes under static condition and up to 62% under continuously flowing condition.

## Introduction

Ultrafiltration (UF) technologies have been used extensively for water treatment applications due to their extraordinary ability to remove particles and microorganisms. UF and other poly(ethersulfone) (PES)-based membrane separation techniques face significant challenges in terms of membrane fouling, where substances accumulate on the membrane surface and/or within its pores^1–3^. Membrane fouling leads to deterioration of performance and increases maintenance and operational costs^4^. There are various types of fouling, each dependent on the nature of the foulant itself, such as scaling, inorganic, organic biofouling, protein, and antifoam^5^. These fouling agents often coalesce and collectively contribute to the degradation of the separation process, causing to a significant decrease in both membrane efficiency and selectivity^6,7^.

Many processes have been applied to mitigate membrane fouling, including physical methods (e.g., backwashing and air spraying), chemical methods (e.g., chemical detergents), and biological methods (e.g., phage and bacterial quorum quenching)^8^. Therefore, pretreatment, hydrodynamic operation parameters, and membrane surface modification together can represent the three basic techniques for achieving an effective low-fouling membrane-based separation process. These three techniques reduce the cost of the filtration process and minimize contaminants in the receiving water bodies^9^.

The initial step in membrane biofouling involves the attachment of microorganisms to the membrane surface^10^. Therefore, an anti-attachment approach is likely to be more effective than antimicrobial approaches that focus solely on killing microorganisms already attached to the membrane^11^. The widespread use of antibiotic-resistant bacteria has made traditional treatments less effective at inhibiting bacterial biofilm formation^12^. Furthermore, if disinfectants kill microorganisms during the pretreatment process, the inactive bacterial biomass may serve as nutrients for the growth of new bacteria^13^. Therefore, pretreatment should include some precautions to avoid possible biofouling, not only to decrease the number of microbial cells but also to prevent the regrowth of bacteria. Thus, free chlorine disinfection is not effective for microbial inhibition in that work due to the bacterial regrowth^14^.

The degree of bacterial attachment/adsorption on a surface is influenced by physicochemical properties, including the cell surface hydrophobicity, charge, roughness, and topography of the surface^15^. Bacteria isolated from the biofilm formed on the membrane surface suggested that hydrophobic interactions could facilitate the initial bacterial attachment step onto the surfaces^6^. Additionally, the type of bacteria, Gram-positive or Gram-negative, should be considered effective features of the bacterial attachment process due to their outer layer structure. Bacterial cells tend to attach more strongly to surfaces that match their own hydrophobicity or hydrophilicity. In general, hydrophobic bacteria adhere more strongly to hydrophobic surfaces, whereas hydrophilic bacteria adhere more strongly to hydrophilic surfaces^16^. *Escherichia coli* generally exhibits hydrophilic characteristics due to its outer membrane components, such as lipopolysaccharides, which contribute to its hydrophilic nature^17^. *Staphylococcus aureus* can adhere to both hydrophobic and hydrophilic surfaces, but it tends to adhere more strongly to hydrophobic surfaces^16^.

According to some researchers, the roughness of membrane’s surface increases the area available for bacterial attachment, thereby inducing biofilm formation^18,19^. Conversely, other studies suggest that membrane roughness combined with hydrophobicity may reduce biofouling by lowering the chances of bacterial attachment and inhibiting biofilm development^20,21^. This phenomenon could be linked to the formation of eddies on the membrane surface, which may disrupt bacterial adherence and enhance self-cleaning properties through the “lotus effect”^19^. The lotus effect describes the superhydrophobic characteristics of lotus leaves, which cause water droplets to bead and roll off, carrying away contaminants and bacteria. Mimicking this effect in membrane-based filtration could lead to surfaces that resist biofouling and enhance membrane durability^22^.

Many researchers are working on surface modification of membrane to reduce bacterial attachment^4,18,23^. PES-based membrane widely used in the food and water filtration applications as ultrafiltraion and nanofiltration membrane because of their outstanding thermal, mechanical properties and heat stability^2,24,25^. Additionally, PES is a widely used support membrane material in reverse osmosis desalination due to its high performance and cost-effectiveness. Numerous modification methods have been reported^26,27^ to reduce PES-based membrane (bio)fouling. PES-based membranes have good strength; excellent oxidative, thermal and hydrolytic stability; high permeability with a relatively low surface energy; and large water contact angles^1,28^. PES properties can be modified to obtain a hydrophilic surface. Surface hydrophilicity and surface structure are considered significant factors for enhancing fouling-repellent surface properties^7,18,25^. Membrane modification is used to enhance the antifouling property of a PES- based membrane and improve its hydrophilicity and filtration performance^4,29^. One of the promoting surface modifications is the grafting of more hydrophilic polymer brushes onto the membrane surface, in which steric hindrance, surface energy, and the osmotic effect of grafted polymer branches cohered to reduce the attachment/adsorption of foulants^18,23,28,30^.

Recently, our team succeeded in modifying PES-based membranes via an enzyme-catalyzed treatment method with the following amine-bearing phenol modifiers: 2-aminophenol (2-AP), 3-aminophenol (3-AP), and 4-aminophenol (4-AP)^30–32^. The reported electrochemical properties of the three positional isomers (ortho, meta, and para) are widely different^33^. The modified membrane surface showed different layer structures and indeed different resistances towards protein adsorption, reaching up to a 90% protein adsorption reduction (as a first step to suppress biofilm growth) without major changes in the main membrane characteristics and structure, as mentioned in^31^.

Aminophenol is a very promising compound because of its two functional groups; the amino and the hydroxyl groups, which can be oxidized^34^. Therefore, aminophenols can exhibit electrochemical behavior and react as both phenols and anilines, which play a vital role in the polymerization process through these two reactive functional groups. Aminophenols were chosen as substrates for the laccase enzyme (extracted from *Trametes versicolor*) under eco-friendly polymerization because their amino groups could add unique properties to PES membranes^31^ through improving the hydrophilicity of the amine and hydroxyl groups and the filtration performance of PES-based membranes^35^.

The -OH, -NO_2_, and -NH- groups exhibited pronounced cytotoxic activity, suggesting that compounds containing these groups may also possess antineoplastic properties^36^. Phenolic compounds can denature bacterial proteins, inhibit bacterial growth at high concentrations, and prevent biofilm formation at lower concentrations^37^. Additionally, phenolic compounds may have the potential to inhibit quorum sensing in certain bacterial species^38^. The antimicrobial activity of phenolic compounds can be represented in many different ways, such as by affecting the permeability of both cell walls and cell membranes, releasing intracellular constituents, and interfering with membrane functions such as electron transport, nutrient uptake, protein and nucleic acid synthesis, and enzyme activity^39^.

Poly(aminophenol) compounds, which contain multiple amines and/or hydroxyl groups, are highly effective in antifouling applications. In particular, poly(3-aminophenol) enhances antifouling performance by effectively inhibiting biofilm formation^40^. Additionally, poly(3-aminophenol) acts as both an antibacterial agent and a corrosion inhibitor^41^. An environmentally friendly enzyme-catalyzed modification for PES-based membranes was achieved by using three aminophenol isomers (2-AP, 3-AP, and 4-AP) in combination with laccase as a biocatalyst^42^. A brush-like poly(3-AP) and pancake combined with a poly(2-AP) layer structure was proposed, as mentioned in our previous work^30,32^, which is effective in improving bacterial anti-attachment in the PES-based membranes^42^. For example, the use of 5 mM 2-AP with a 60-minute modification at 25 °C led to improvements of up to 15.4% in water flux and 81.27% in protein repellence^31^. The contact angle of the unmodified spin-coated PES silicon wafer was reduced from 78.8° ± 1° to 63.4° ± 1° with modification, indicating an increase in hydrophilicity with modification^32^.

However, 4-AP causes oxidative stress in bacteria because of the hydroxyl groups (phenolic characteristic) on the aminophenol, which can play an antibacterial role^29^. The best protein repellence was obtained with membranes modified with a low modifier concentration; membrane modified using 5 mM 4-AP for 120 min at 25 °C, resulting in up to a 90.8% reduction in protein adsorption^31,43^. In the case of 4-AP modification, the resulting modification layer is likely composed of hydroquinone dimers, and the formation of poly(4-aminophenol) has not been suggested. The use of a low concentration of 4-AP resulted in an effective anti-bacterial (not anti-attachment) structure layer. In other words, a high concentration of 4-AP resulted in a thicker pancake layer with less effective either anti-attachment or anti-bacterial modification layer that resulted in a membrane with lower antifouling efficiency. Compared with that of the unmodified membrane, the clean water flux increased up to 17.7%. In the case of the 3-AP-modified membranes, the water flux increased up to 35%, whereas protein adsorption decreased to 90%. The permeability of 3-AP-modified membranes has clearly improved because amino groups can enhance the hydrophilicity of the membrane surface^44^. In the brush-like oligomer layer of 3-AP, there were free amine groups and hydroxyl groups in the produced layer. The branched brush-like oligomer chains with poor miscibility resulted in good foulant repellence. Compared with that of the unmodified PES layer, the static water contact angle on the 3-AP-modified PES layers (surfaces) was dramatically reduced by up to 44%^30^.

The effectiveness of these modified membranes has been confirmed through a comparative study between conventional chlorination pretreatment and a novel enzyme-catalyzed modification of PES with 3-AP as an efficient strategy for reducing bacterial attachment^45^. This approach avoids the use of timed antimicrobial agents that can potentially promote bacterial regrowth^13^. The results of this study revealed that the modified PES-based ultrafiltration (UF) membrane, without the need for prechlorination, maintained the highest initial flux (3.27 ± 0.13 m^3^ m^−2^ h^−1^) and exhibited a water productivity that was 1.5 times greater than that of the unmodified membrane. In our previous studies, we examined protein fouling, and the modified PES-based membranes we developed effectively repelled protein adsorption. This was achieved through steric hindrance and the osmotic effect of the hydrated grafted oligomer chain on the PES-based membrane surface. This study is a complementary study for our research which concerning the microbial point of view, and all physical characterizations of the modified membrane have been mentioned in our previous published researches^30–32^ in detail. Both unmodified and modified membranes were characterized using X-ray diffraction patterns (XRD), Fourier transform infrared spectroscopy (FTIR), Proton nuclear magnetic resonance (^1^H-NMR), scanning electron microscope (SEM) as well as mechanical strength testing. Moreover, the shape and structure of the modified layer(s) grafted on PES surface were investigated using other analytical techniques that include static water contact angle and scanning probe microscope (SPM) images.

In the present study, our focus has shifted to evaluating the anti-attachment and antimicrobial properties of these modifying layers and testing their performance under both static and continuously flowing conditions. Understanding their performance under both static and continuously flowing conditions is crucial. Static conditions were shown to result in a high bacterial load on the modified membranes. *Escherichia coli* (*E. coli*) and *Staphylococcus aureus* (*S. aureus*) were selected as model organisms representing Gram-negative and Gram-positive bacteria, respectively, and all safety precautions were taken carefully. *S. aureus* is widely applied because of its clinical importance, well-studied characterization, and ability to mitigate antibiotics. *S. aureus* is a coccus Gram-positive bacterium that is 1 μm in diameter and small in size and forms biofilms, which are important in chronic infections^46^. Scanning electron microscopy (SEM) was employed to examine the attached bacterial cells on the PES-based membranes and surfaces. Additionally, the minimum inhibitory concentration (MIC) of the three aminophenol modifier isomers against both *E. coli* and *S. aureus* were determined. Overall, this work aims to elucidate how the incorporation of the selected modifying layers influences bacterial attachment and growth on PES-based membranes, which has significant implications for the development of antifouling and antimicrobial membrane technologies.

## Materials and methods

### Materials

Aminophenols; 2-Aminophenol (2-AP, 99.5%), 3-aminophenol (3-AP, 99.8%), 4-aminophenol (4-AP, 99%), laccase from *Trametes versicolor* (> 0.5 U/ml), acetic acid (99%), sodium acetate (anhydrous, 99%), and dichloromethane (DCM, 99.9%) were obtained from Sigma‒Aldrich. Sodium phosphate monobasic and disodium hydrogen phosphate 2-hydrate (extra pure) were obtained from Riedel-de Haen (USA). Ethanol (analytical reagent grade) was obtained from Fisher. A flat sheet of commercial organic poly(ethersulfone) (PES; 0.03 μm pore size) was obtained from Sterlitch (USA). The poly(ethersulfone) (PES) polymer was obtained from BASF (Germany), and prime-grade silicon wafers with a 2.5 nm native oxide layer were purchased from wafer Net, Inc. (USA). Tryptone was obtained from CONDA. Yeast extract was obtained from Bio Basic Canada INC. Sodium chloride (NaCl) was obtained from MP BioMedicals.

### Microorganism suspension/culture media

*Escherichia coli* NCTC10418 and *Staphylococcus aureus* ATCC6538 strains, laboratory bacterial strains, were used to represent for Gram-negative and Gram-positive bacteria, respectively. Luria–Bertani (LB) liquid medium was prepared. Phosphate buffer saline (0.1 M PBS, pH 7) was used for serial dilutions. Overnight bacterial cultures were prepared by inoculating 5 ml of LB broth with stock cultures of both strains separately and then incubating them in a shaker incubator at 150 rpm at 30 °C for 24 h. A total of 10 ml of bacterial culture with an OD_600_ of approximately 1 was centrifuged at 6000 rpm for 15 min. Then, the supernatant was discarded, and the pellet was suspended in 1 ml of fresh LB broth to concentrate the bacterial suspension stock ten times and prepare the concentrated bacterial culture.

### Determination of the minimum inhibitory concentration (MIC)

To study the inhibitory effect of the isomers used on bacterial growth, the minimum inhibitory concentration^36^ was determined using the monomers (i.e., modifiers). Different concentrations of each aminophenol monomer were prepared (5, 10, 15, 20 mM) in 25 ml of sterile LB media and then inoculated with 12.5 µl of the concentrated bacterial culture with an OD = 1 ± 0.18 of its 10^−1^ dilution as previously described. The cultures of *E. coli* and *S. aureus* were incubated in a shaker incubator at 30 °C for 24 h with stirring at 150 rpm. After incubation, the first test tube with no visible growth of the microorganism was taken to determine the MIC value of the sample. Subsequently, serial dilutions in PBS were prepared, and 100 µl of the suitable dilution was plated on agar plates (3 replicates of each concentration for each aminophenol monomer were inoculated). The plates were incubated overnight at 30 °C, and colonies were counted.

### Modification of the poly(ethersulfone) (PES) membrane surfaces

#### Spin-coating with poly(ethersulfone) for silicon wafer surfaces

Silicon wafers with a silicon dioxide top layer of approximately 70 nm were cut into strips of 1.6 × 5.5 cm. The strips were sonicated in ethanol 70% for 10 min and dried under a flow of nitrogen gas. The strips were subsequently sterilized and cleaned by a UV lamp for 15 min followed by a flow of nitrogen gas. After that, the cleaned strips were spin-coated with 0.5 wt% PES solution in dichloromethane for 30 s at 5000 rpm. The spin-coated PES strips were kept in an oven at 200 °C for 1 h for drying^30,43^.

#### Modification of PES membrane surfaces

**Fig. 1:**
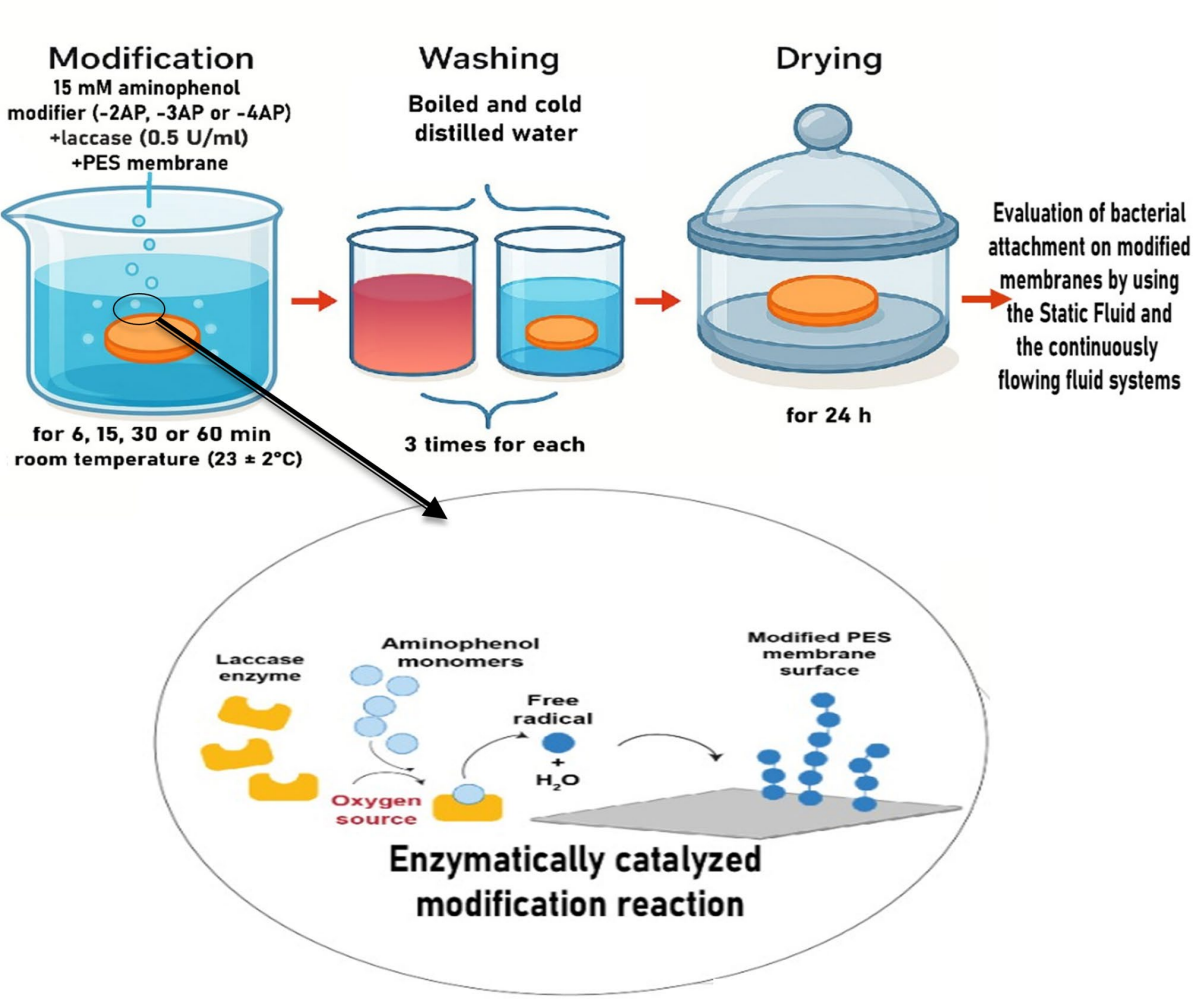
A schematic diagram illustrating the modification process of poly(ethersulfone) (PES) membrane with an enlargement clarifying the enzymatically catalysed modification reaction of the membrane (created with the assistance of www.BioRender.com).

The flat sheets of commercial PES membranes were cut into circles with a diameter of 4.5 cm to be used in bacterial attachment investigations under a static fluid system, and three membrane samples were used for each modifier. Both PES commercial membrane circles and spin-coated PES strips were immersed in 40 ml of 0.1 M sodium acetate buffer, pH 5, containing 15 mM of a specific aminophenol modifier (2-AP, 3-AP, or 4-AP) and laccase enzyme (0.5 U/ml). Air was bubbled through the solution for mixing and as a source of oxygen for the enzymatic reaction as shown in Fig. 1. The reaction was carried out at room temperature (23 ± 2 °C). After the specific modification time (6, 15, 30, or 60 min), the PES membranes/strips were washed by immersion in boiled distilled water three times and then by immersion with cold distilled water three times, after which the samples were dried for 24 h in glass-covered dishes in desiccator^30,31,45^.

### Evaluation of bacterial attachment on membranes using the static fluid system

Both unmodified and modified commercial membranes were placed in glass Petri dishes (10 cm). Each half-membrane circle was immersed in 20 mL of phosphate buffer saline (0.1 M PBS, pH 7), which was inoculated with 10 µl of a concentrated bacterial suspension of *E. coli and S. aureus* with an OD_600_ of 1 ± 0.20 at the 10^−1^ dilution. The immersion time was 2.5 h at room temperature (23 ± 2 °C) After immersion, the membrane halves were washed with distilled water twice by shaking at 120 rpm for 15 min and then incubated in a shaker incubator at 30 °C for 24 h at 150 rpm in phosphate buffer to detach the attached bacterial cells from the membranes^43^. Subsequently, 100 µl of diluted 10^−3^ bacterial suspension was plated on Luria–Bertani (LB) agar plates, and the process was repeated three times. The plates were incubated for 24 h at 30 °C, after which the number of colonies was counted as CFU/cm^2^. For each modification condition, the test was repeated twice on different days by using two independently prepared bacterial cultures.

### Evaluation of bacterial attachment on membranes using the continuously flowing fluid system

This experiment was investigated using a flow cell connected to a pump. Unmodified and modified spin-coated PES strips of silicon wafers were placed on the sample support inside the flow cell. The bacterial suspension was diluted in 500 ml 0.1 M PBS pH 7 (250 µl of bacterial suspension of *E. coli and S. aureus* OD_600_ = 1 ± 0.16 of its 10^−1^ dilution). First, the system was washed with ethanol 70% for 15 min to be sterilized. The sterile flow fluid system was subsequently washed with sterile distilled water for 15 min to remove any traces of ethanol 70% before the spin-coated PES strips of silicon wafers were used for testing. The system was filled with the bacterial suspension, which was discarded. The bacterial suspension was circulated through the system for 2.5 h at a flow rate of 35 ml/min. Modified and unmodified PES slides of silicon wafers were washed by immersion three times in fresh 80 ml of sterile distilled water, incubated in 30 ml of sterile PBS and left in a shaker incubator at 120 rpm at 30 °C for 24 h to collect the attached bacterial cells to model spin-coated PES slides of silicon wafers. Then, 100 µl from 10^−3^ dilutions were plated on LB agar plates in triplicate, the plates were incubated for 24 h at 30 °C, and the number of colonies was counted.

### Scanning electron microscopy (SEM) imaging

The commercial PES membrane/spin-coated PES surfaces with attached *E. coli and S. aureus* bacteria were examined by SEM imaging. The attached bacterial cells were fixed in a solution (2.5% glutaraldehyde, 4% formaldehyde in 0.1 M phosphate buffer; pH 7.2)^47^ and serially dehydrated in ethyl alcohol. After critical-point drying, scanning electron microscopy can be used to image the samples without damage under vacuum. PES membranes/surfaces were coated with gold via a sputter-coating system (Ion sputtering device, JEOL, JFC-1100E, Japan) and then examined via SEM (JEOL, JSM-5300, Japan) at various resolution powers operating at 20 kV^45^.

### Statistical analysis

Data analysis was executed using Statistix 10.0 (USA) software. The effects of the three isomers, with different modification time, on membrane biofouling were evaluated based on the completely randomized design (CRD) with two response variables: total plate count (TPC) of *E. coli* and *S. aureus*. All experiments were conducted in triplicate for each trial, and data was reported as mean ± standard deviation. The confidence interval of statistical significance was defined at 95% (*P* ≤ 0.05).

## Results and discussion

This work aimed to investigate the anti-attachment properties of three isomers of aminophenol against *E. coli* and *S. aureus*, which are Gram-negative and Gram-positive bacteria, respectively using both static and continuously flowing fluid systems.

### Minimum inhibitory concentration of the isomers of aminophenol against *E. coli* and *S. aureus*

**Fig. 2:**
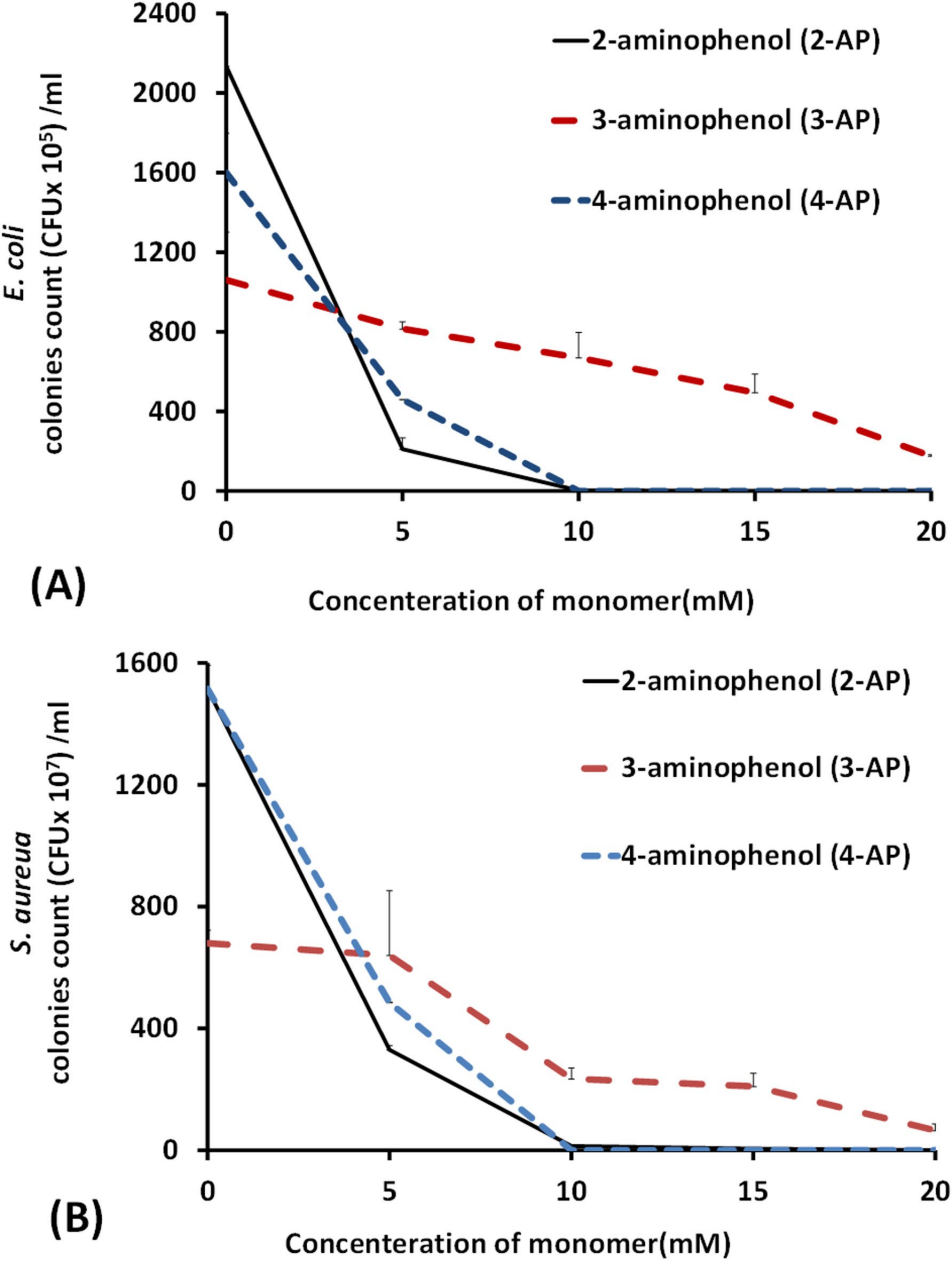
Bacterial counts of MIC for the modifiers/monomers (2-AP, 3-AP and 4-AP) concentration against (**A**) *S. aureus* and (**B**) *E. coli* strains.

To investigate the antibacterial effects of the three isomers of aminophenol, the minimum inhibitory concentration was determined. As shown in Fig. 2, the observed difference in antibacterial efficacy between 3-aminophenol (3-AP) and 2-aminophenol (2-AP) can be attributed to their structural differences and mechanisms of action. 3-AP, with the hydroxyl group at the meta position, may have reduced membrane penetration and lower reactive oxygen species (ROS) generation, resulting in its weak antibacterial effect at 10 mM. In contrast, 2-AP’s structural configuration enhances its ability to disrupt bacterial metabolic processes and induce oxidative stress, making it more effective against both *E. coli* and *S. aureus*^32^. This highlights the importance of chemical structure in the antibacterial activity of aminophenol isomers. On the other hand, as noticed in Fig. 2, both 2-AP and 4-AP isomers can kill both *E. coli* and *S. aureus* strains at a 10 mM monomer concentration. Although 3-AP has a weak effect on killing bacterial cells, it can also decrease the number of counted bacteria by increasing its concentration. Notably, the determined performance is due to the effect of the modifier as a monomer, and this inhibition effect could be diminished as oligomers or polymers are grafted onto the membrane surface through the oxygen of the phenol group, with its effect diminishing as antibacterial moieties. Therefore, the antibacterial efficiency of aminophenol polymers should be determined because they are formed mostly through covalent bonds during polymerization, which most likely occur through the phenolic oxygen of the hydroxyl group, with the phenolic lethal effect disappearing as bactericidal compounds^29,32^.

Aminophenols are known to have antimicrobial activity^29^, and 4-aminophenol (4-AP) has shown strong bactericidal activity against two types of bacteria: *S. aureus* and *E. coli*. The cells of *E. coli* or *S. aureus* treated with 10 mM 4-AP is not exist completely from the counted plates, which is in full agreement with many studies^29,48,49^. The greater bactericidal activity of aminophenol (especially 4-AP) may be because aminophenol can more readily diffuse across the bacterial cell walls and the cytoplasmic membrane of bacteria^29,48^. The active phenolic compounds might have several targets to inhibit bacterial growth. They not only attack cell walls and cell membranes but also interfere with membrane functions including electron transport, nutrient uptake, protein, nucleic acid synthesis, and enzyme activity^39^. This fully agrees with previous work and suggests that 4-AP does not form a poly(4-AP) polymeric layer. Nevertheless, it forms monomers or dimers of quinone and hydroxyl groups that maintain its antibacterial phenolic characteristics^32^.

### Evaluation of bacterial attachment by using a static fluid system

A clear trend was observed when *E. coli* was used against 2-AP modified PES membrane, and increasing the modification time resulted in a continuous reduction in the number of counted *E. coli* cells to approximately 80% at the 60 min modification time. At the 6 min treatment time, the layer was formed by very short oligomers that could not inhibit bacterial attachment very well, as mentioned in our previous work^31^ but it can show a good bacterial growth inhibition due to its phenolic functional group. An increase in the modification time to 15 min may increase the length of the grafted oligomers, resulting in an improvement in protein repellence relative to that of the unmodified membrane, as previously reported^32^.

**Fig. 3:**
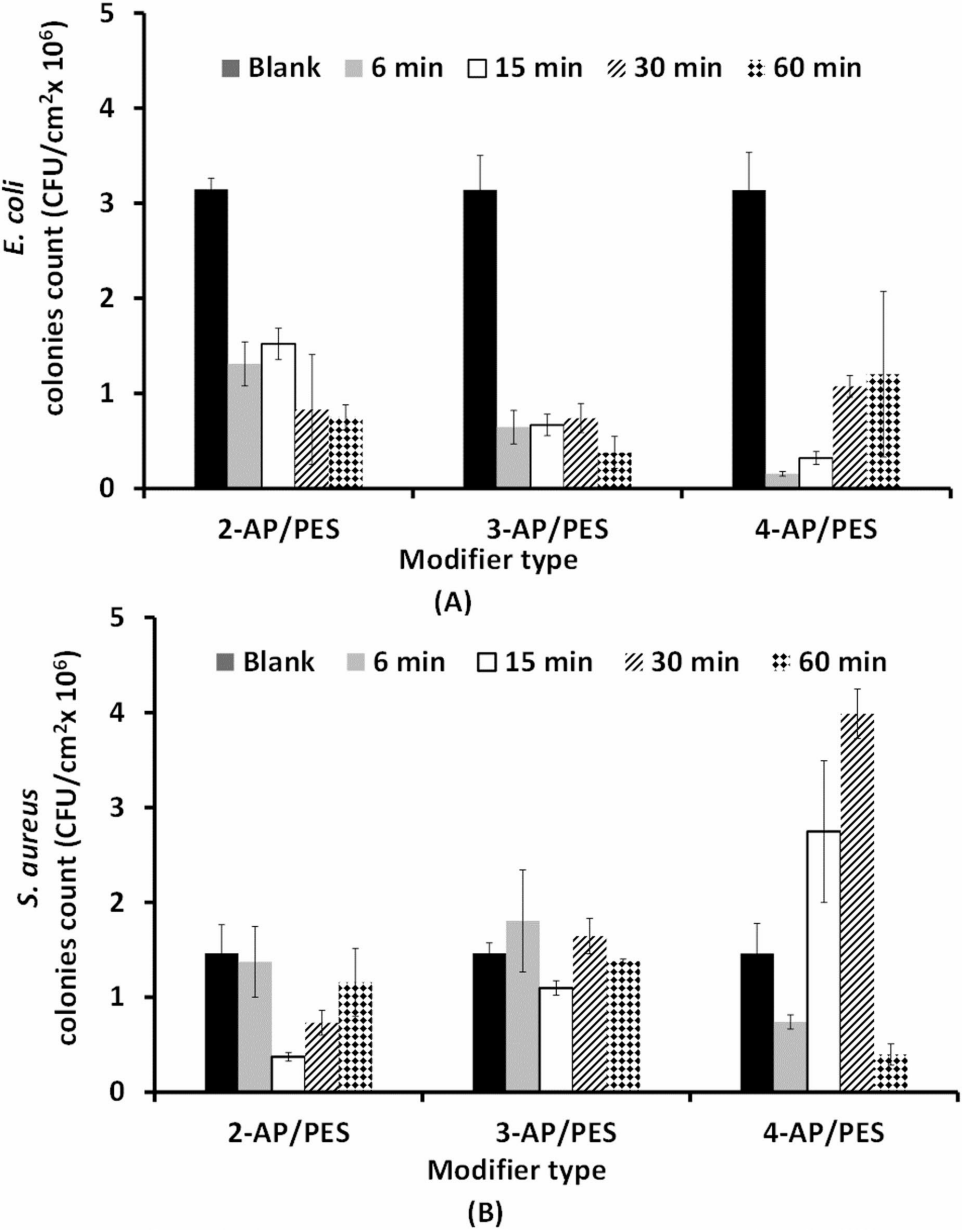
The bacterial counts on the modified membranes as a function of the modifiers used (2-AP, 3-AP, and 4-AP), and different times (6, 15, 30, and 60 min), Blank is unmodified membrane. The test was performed under static conditions with commercial PES membranes, (**A**) *E. coli* and (**B**) *S. aureus.*

On the other hand, for poly(2-AP) (Fig. 3) there was no specific trend in the case of *S. aureus*, although the most interesting condition was modification for 15 min, which reduced the number of attached bacterial cells to 75% compared with that of the unmodified membrane. *S. aureus* is a Gram-positive, round-shaped bacterium that has a moderately negative net charge at neutral pH. In Gram-positive bacteria, the reason for this negative charge is the presence of teichoic acids linked to either the peptidoglycan thick layer or the underlying plasma membrane. These teichoic acids are negatively charged because of the presence of phosphate in their structure^50^.

In addition, the 2-AP monomer has a good bactericidal effect on both *E. coli* and *S. aureus*, as recorded in the MIC experiments and supported by the data of other researches^37,51^. The greater effect of poly(2-AP) on *E. coli* than on *S. aureus* (Fig. 3) can be attributed to the more negatively charged *E. coli* bacterial cells being affected by more repulsion forces with the negatively charged modified membrane, resulting in greater inhibition of bacterial attachment, as mentioned by Sonohara, et al.^50^. Gram-negative bacteria have an outer covering of lipopolysaccharides that impart a strong negative charge to the surface of bacterial cells and help maintain the integrity and stability of the bacterial outer membrane. Lipopolysaccharides act as barriers against harmful substances, including antibiotics and detergents^50^. On the other hand, another study on the effects of the surface charge and hydrophobicity of *E. coli* on its adhesion to beef muscle illustrated that the *E. coli* strain is moderately hydrophobic. However, at relatively high modification times, excess 2-AP monomers can react with each other, forming homopolymers, as well as bonding to the membrane surface, which can minimize the repulsion forces between living cells and the membrane surface^32^. Additionally, the increase in the modification time may cause the formation of a pancake layer^32^ that diminishes the steric hindrance effect of brush-like oligomers formed at a shorter modification time. Therefore, a low grafting yield may result in better flux and better protein repellence than a high grafting yield^52^.

**Fig. 4:**
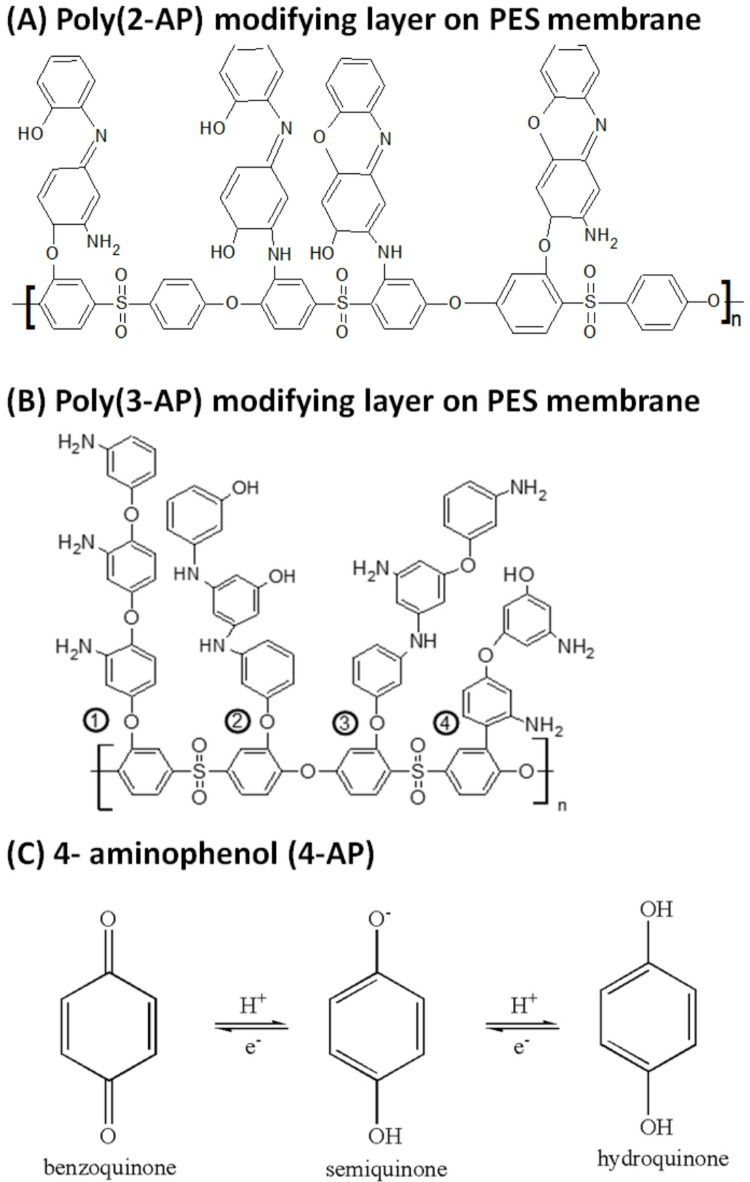
The chemical structures of the proposed modifier layers created on the surface of poly(ethersulfone) (PES) membranes using different aminophenol isomers: (**A**) poly(2-aminophenol)^32^, (**B**) poly(3-aminophenol)^30^, and (**C**) 4-aminophenol conjugate^54^.

The same no obviously trend was obtained performance of poly(2-AP) modification was obtained with the poly(3-AP) modifier layer. Moreover, *S. aureus* cells reduced by approximately 25% in response to the addition of poly(3-AP) after 15 min of modification. There was no clear difference in the count of *E. coli* cells with different numbers of poly(3-AP) modifications, and all the reduction were approximately 90%. The bacterial attachment inhibition of the poly(3-AP) layer after 15 min of modification depends on its brush-like surface as shown in Fig. 4, which results in good steric hindrance and hydrophilicity of the membrane layer. By increasing the modification time, the bacterial attachment inhibition ability may decrease due to the cross-linking of the brush-like structure, which decreases its protein repellence ability as well^30^. The weak bactericidal effect of the 3-AP monomer, as shown in the MIC experiment in Fig. 2, may have been a vital factor in its weak inhibition of *S. aureus* attachment. These interfered chains may entrap protein molecules, resulting in increased protein adsorption at high grafting yields^31^. However, poly(3-AP) antibacterial activity has shown a good response against *S. aureus* and *E. coli*, in other study^49^.

In the case of the 4-aminophenol modifier (4-AP), the *S. aureus* counts were slightly greater than those of the unmodified membrane, especially after 15 min and 30 min of modification. With shorter modification time (6 min), the reduction in the bacterial count was 60%, while the number of cells at 60 min was lower than the number of cells attached to the unmodified membrane. Moreover, the 4-AP modification caused the greatest reduction in *E. coli* count by more than 95% with a 6 min treatment time, and the number of counted cells increased with increasing the time up to 60 min (approximately 60% reduction). The oligomers of 4-AP has chemical oxidative properties that can inhibit the growth of both *S. aureus* and *E. coli* bacterial strains at different levels^49^ and has significant protein repellence (up to 90.8%) while keeping the bulk properties of the base membrane intact^31^. The significant reduction in the number of counted cells, especially at very short modification times, can be attributed to the tendency to form quinone, which seems to prolong the lag phase of microbial growth and increase the death rate^53^.

On conclusion, the observed increase in *S. aureus* counts at 15 and 30 min, followed by an acceleration at 60 min, is attributed to the oligomers of 4-aminophenol. These oligomers maintain their quinonoid structure and toxic phenolic characteristics, leading to the formation of three-dimensional adsorbed layers. In many cases, these results in an increase in adsorbed layer thickness, which may have an uncontrolled trend effect on both anti-attachment and anti-bacterial properties. This interplay can explain the fluctuations in bacterial counts observed over time. A similar fatal bactericidal effect was observed in the MIC test of the 4-AP monomer in both *S. aureus* and *E. coli* bacterial cells (Fig. 2).

**Fig. 5:**
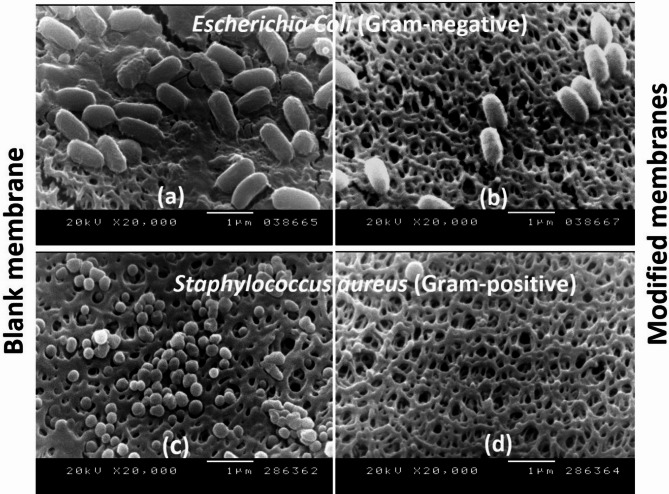
SEM images of unmodified PES membranes with attached *E. coli* (**a**) and *S. aureus* strains (**c**) and modified PES membranes [15 mM 3-AP, 15 min modification time, 0.1 M sodium acetate buffer at pH 5 at 25 °C] with attached *E. coli* (**b**) and *S. aureus* strains (**d**).

Scanning electron microscopy (SEM) imaging of the unmodified and modified membranes with 3-AP confirmed a reduction in the number of attached bacterial cells for both the *E. coli* and *S. aureus* strains (Fig. 5). Therefore, the effect of steric hindrance when 3-AP is used is more pronounced than that when 2-AP and 4-AP modifiers are used, especially in the case of *E. coli*. A mucilage-secreted layer could be observed in the attached *E. coli* on the unmodified membrane image, which disappeared not exist on the poly(3-AP)-modified membrane. This mucilage secretion is produced to protect the bacterial community and form a “biofilm”^55^. This disappearance of the mucilage layer in the SEM image of the poly(3-AP)-modified membrane is likely due to the inhibitory effect on biofilm formation^51^. There is an adhesive interaction resulting from the sticky nature of the protection layer and the appendages of some microorganisms, which can help the cell anchor to the membrane surface^56^. Some researchers have reported that typical mucilage secretions are generally negatively charged and that many polysaccharides can form extensive cross-linked structures that trap some foulants. In addition, the modified PES membrane using layer of 4-hydroxybenzoic acid clearly has potential for the reduction of polysaccharides, which could be related to the surface morphology^52^.

Some researchers have suggested that at short reaction times, the surface becomes grafted, leading to a thin layer that is more hydrophilic than the unmodified PES membrane^52^. This layer effectively reduces the strong adsorption of proteins, polysaccharides, and polyphenols. The reduction in the number of attached bacterial cells relative to the number of bacteria that can adhere to and grow on the unmodified PES membrane surface can most likely be attributed to the brush-like structure of the poly(3-AP) modification layer^45^. The effectiveness of these structures against bacterial attachment is caused by steric hindrance that keeps the bacterial cells at a distance from the surface, which results in the weakening of van der Waals interactions, which helps in biofilm inhibition and prevents secretions^43^. In addition, the hydrophilicity of the modified membrane reduces the number of bacteria attached to the membrane surface^4,55^.

The effect on *E. coli* was more pronounced than its effect on *S. aureus*. This may be attributed to the repulsion between the charged groups on the modifying layer and the outer cell membrane charge of *E. coli*. This is in addition to the steric hindrance of the formed brush-like modifying layers in the case of the 2-AP modifier at a very short modification time and in the case of the 3-AP modifier, as confirmed by atomic force microscopy (AFM)^30,32^. The modification with 4-AP after 6 min resulted in the greatest *E. coli* bacterial growth inhibition due to the fatal effect of the formed quinone, which diminished with increasing modification time and the formation of a pancake layer of quinone oligomers^31^.

### Bacterial attachment evaluation by using the continuously flowing fluid system

**Fig. 6:**
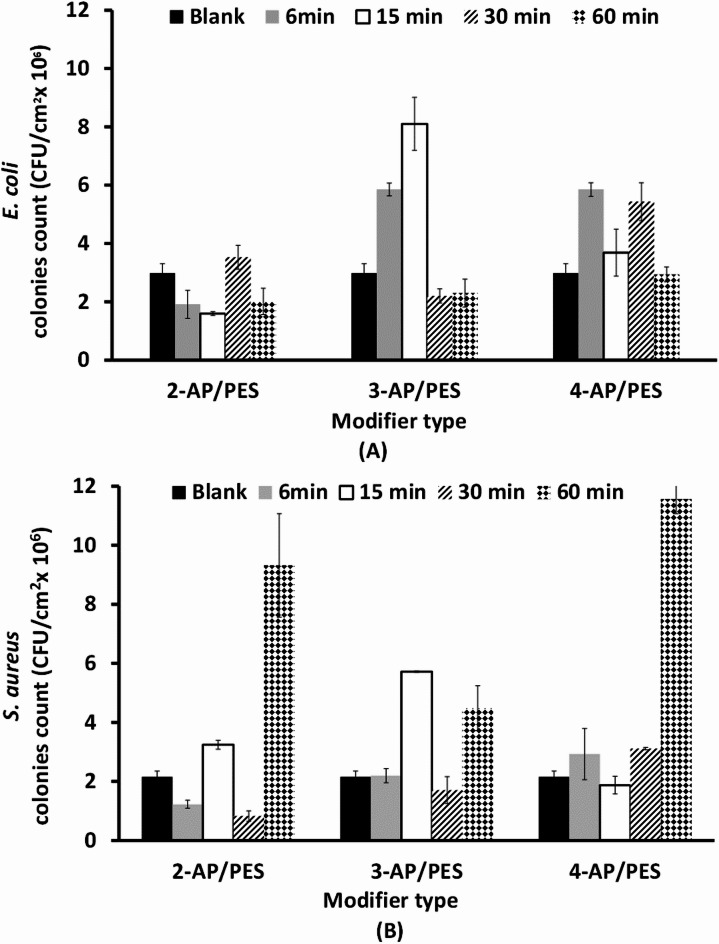
The bacterial counts on the modified spin-coated PES surfaces as a function of the modifier used (2-AP, 3-AP, and 4-AP). Different modification times (B, 6, 15, 30, and 60 min) were used in a continuously flowing fluid, and both (**A**) *E. coli* and (**B**) *S. aureus* strains were tested.

The spin-coated PES surface modified by the poly(3-AP) layer for a 30 min modification time was the minimum required time to form a brush-like layer that can resist bacterial attachment. For the general trend, we can conclude that the modification using a 3-AP modifier for 30 min resulted in a reduction of 26% in the number of attached *E. coli* cells. When *E. coli* was tested, as shown in Fig. 6, there was no clear trend of decreasing or increasing numbers of attached bacteria. In the case of spin-coated PES surfaces modified with poly(2-AP), the combination of brush-like and pancake surfaces may be the reason for the response to cell anti-attachment as mentioned by Nady in^32^, such as a 36% reduction in the number of attached *E. coli* cells on the modified spin-coated PES surface for 6 min.

The case of 4-AP is not yet understood, but indeed, modification with 4-AP does not affect bacterial attachment in the case of continuously flowing fluid rather than static fluid. This may be explained by the washing out of the settled quinone or free 4-AP monomer on the modified PES surface membrane, which had no effect on the number of attached bacterial cells.

**Fig. 7:**
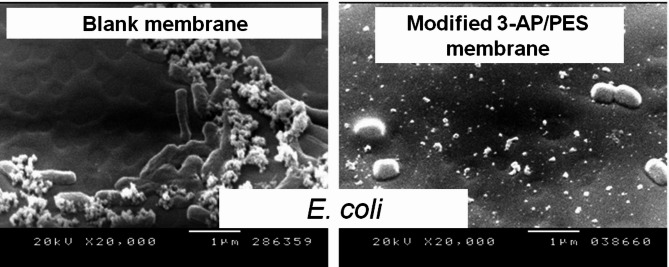
SEM images of *E. coli* attached to both unmodified and modified spin-coated PES strips on silicon wafers [15 mM 3-AP, 15 min modification time, 0.1 M sodium acetate buffer at pH 5 at 25 °C].

In general, the three modifiers led to a gradual decrease in the number of attached cells until the 30 min modification time, after which these numbers significantly increased with the 60 min modification time, as shown in Fig. 6. For example, modification with 2-AP for 6 min resulted in a 43% reduction in the number of attached.

*S. aureus*, whereas this percentage increased to 62% after 30 min of modification. SEM examination of poly(2-AP) revealed the disappearance of the sharp edges of the pores of the unmodified PES layer, which decreased the bacterial attachment ability, in agreement with Nady^32^. This is accompanied by a reduction in the modifying layer shape/roughness with no effect on the pore size of the membranes, and this layer appears pancake shaped with a longer modification time.

It was reported that the bactericidal activity of grafted 4–AP against *E*. *coli* is slightly lower than that against.

*S. aureus*, suggesting that the oligomer may have more problems affecting or attacking the cell wall of the Gram-negative bacteria *E*. *coli* because of the outer membrane barrier^48^. In addition, the appendages of the microorganism’s surface, which may be present on the *E. coli* surface, can remove the water film between the cell of the microorganism and the membrane surface to allow the cell to adhere to the surface directly, as shown in Fig. 7, which was also mentioned in the studies of Al-Juboori and Yusaf^56^. To completely understand the observed behavior, the zeta-potential of the modified surface should be determined, so that further research is needed. However, the modified surfaces resulted in a reduction in bacterial attachment (to various degrees). At long modification times, bacterial growth increases because of a non-uniformly formed modification layer with cross-linking, and shield bacterial cells^52^.

The movement of a bacterial suspension can prevent bacteria from settling in the modified layers of pores and grooves. While the repellent effect on microorganisms may be reduced, it is important to remember that these modified layers still repel both Gram-positive and Gram-negative bacteria. The flow of a bacterial suspension can prevent or slow the adhesion of living cells, allowing the removal of loosely attached bacteria. Conversely, the convective flow of the filtration membranes can actually enhance the deposition of microorganisms on the membrane surfaces. Additionally, when microorganisms approach the membrane surface, their adhesion is facilitated by interactions between the surface and the cell membrane.

It is important to compare the recyclability and sustainability of this novel anti-biofouling method with other existing membrane modification techniques. One key advantage of the enzyme-catalyzed modification with 3-AP described in this work is its potential for enhanced recyclability and reusability compared to conventional membrane treatments. Many traditional anti-fouling strategies, such as chlorination pretreatments, rely on harsh chemicals that can degrade the membrane material over repeated cleaning cycles, as shown in previous work [ref]. Additionally, our focus in this manuscript is on studying biofouling inhibition by impeding bacterial attachment on PES membranes through surface modification, which increases the durability and reusability of the PES membranes as well.

## Conclusions

This study investigated the impact of various modifying layers on commercial poly(ethersulfone) (PES) membranes and PES spin-coated silicon wafer surfaces. Specifically, we evaluated the effects of three different isomers of aminophenol modifiers on bacterial attachment using both static and continuously flowing fluid systems. The polymerization process utilizes the oxygen present in the phenolic groups, thereby neutralizing the toxicity of the phenolic monomer within the resulting coated polymer layer. Notably, however, the dimeric/oligomers form of 4-AP may still exhibit certain toxic effects.

This work revealed a reduction in bacterial attachment of up to 90% in the static fluid system and up to 62% in the continuously flowing fluid system. The steric hindrance of the brush-like structure modification layer increases the hydrophilicity of the membrane and reduces the number of attached bacteria, especially when a static fluid system is used. The 4-AP monomer has the strongest bactericidal effect on Gram-positive and Gram-negative bacterial cells at a concentration of 10 mM because of its phenolic lethal effect. The flow motion may force the foulants, including bacteria, to impede and attach to the modified layer pores and grove more than they do when the static system is used. The most promising modification conditions for impeding biofouling in a continuously flowing fluid state were the use of PES spin-coated samples modified with 2-AP or 3-AP for 30 min and the use of PES spin-coated samples modified with 4-AP for 6 min.

Comparing the recyclability and sustainability of this novel antibiofouling approach with those of other existing membrane modification techniques is important. By rejuvenating the antibiofouling membrane strategy, the lifetime of the membrane can be extended. Additionally, avoiding antimicrobial agents in favour of more passive, protein-resistant surface modifications is an environmentally friendly approach that differentiates this approach from alternative antifouling strategies. This helps minimize the risk of promoting antibiotic resistance or causing other unintended ecological impacts. Overall, this antibiofouling system’s recyclability and sustainable design appear to be a notable strength compared with many conventional membrane modification techniques. Further investigation into the long-term durability and reusability of these antibiofouling membranes will be crucial and beneficial for their commercialization.

## Data Availability

All data for this article, including tables and figures, are included in the manuscript.
